# Ionic Mechanisms Underlying the Excitatory Effect of Orexin on Rat Subthalamic Nucleus Neurons

**DOI:** 10.3389/fncel.2019.00153

**Published:** 2019-04-24

**Authors:** Guang-Ying Li, Qian-Xing Zhuang, Xiao-Yang Zhang, Jian-Jun Wang, Jing-Ning Zhu

**Affiliations:** State Key Laboratory of Pharmaceutical Biotechnology and Department of Physiology, School of Life Sciences, Nanjing University, Nanjing, China

**Keywords:** orexin, subthalamic nucleus, motor control, neuronal excitability, ionic mechanisms

## Abstract

Central orexinergic system deficiency results in cataplexy, a motor deficit characterized with a sudden loss of muscle tone, highlighting a direct modulatory role of orexin in motor control. However, the neural mechanisms underlying the regulation of orexin on motor function are still largely unknown. The subthalamic nucleus (STN), the only excitatory structure of the basal ganglia, holds a key position in the basal ganglia circuitry and motor control. Previous study has revealed a wide distribution of orexinergic fibers as well as orexin receptors in the basal ganglia including the STN. Therefore, in the present study, by using whole-cell patch clamp recording and immunostaining techniques, the direct effect of orexin on the STN neurons in brain slices, especially the underlying receptor and ionic mechanisms, were investigated. Our results show that orexin-A elicits an excitatory effect on STN neurons in rats. Tetrodotoxin (TTX) does not block the orexin-induced excitation on STN neurons, suggesting a direct postsynaptic action of the neuropeptide. The orexin-A-induced inward current on STN neurons is mediated by the activation of both OX1 and OX2 receptors. Immunofluorescence result shows that OX1 and OX2 receptors are co-expressed and co-localized in STN neurons. Furthermore, Na^+^-Ca^2+^ exchangers (NCXs) and inward rectifier K^+^ channels co-mediate the excitatory effect of orexin-A on STN neurons. These results demonstrate a dual receptor in conjunction with the downstream ionic mechanisms underlying the excitatory action of orexin on STN neurons, suggesting a potential modulation of the central orexinergic system on basal ganglia circuitry as well as its related motor control and motor diseases.

## Introduction

Orexin (also known as hypocretin) is a neuropeptide first identified in 1998 (de Lecea et al., 1998; Sakurai et al., 1998). There are two splice variants of orexin, orexin-A and orexin-B, both of which exert their physiological functions *via* two types of G-protein coupled receptors, OX1 and OX2 receptors (Tyree et al., 2018). Orexin-A binds to both receptor subtypes with approximately equal affinity, whilst orexin-B shows a 10-fold selectivity for OX2 receptor (Zhang et al., 2013). In the central nervous system, orexin receptors produce excitation by postsynaptic depolarization *via* activation of non-selective cation channels, inhibition of K^+^ channels and activation of Na^+^-Ca^2+^ exchangers (NCXs), as well as presynaptic action through regulation of the release of other neurotransmitters (Kukkonen and Leonard, 2014; Leonard and Kukkonen, 2014). Although originating exclusively from the lateral hypothalamus/perifornical area, the central orexinergic system projects widely throughout almost the whole brain (Broberger et al., 1998; Peyron et al., 1998; Cutler et al., 1999). Accumulating studies have revealed that the central orexinergic system plays a key position in many basic physiological functions, including the sleep-wakefulness cycle, feeding, energy homeostasis and reward processes (Sakurai, 2007; Matsuki and Sakurai, 2008; Zhang et al., 2013; Giardino et al., 2018). Intriguingly, deficit in the orexinergic system in animals and humans results in cataplexy, a motor dysfunction characterized by sudden loss of muscle tone (Chemelli et al., 1999; Sakurai, 2007). The phenotype indicates that orexin may be directly involved in the somatic motor control. However, the knowledge about orexinergic modulation on motor control is still limited.

The basal ganglia is an essential subcortical center responsible for motor initiation and motor learning, within which the subthalamic nucleus (STN) is the sole structure mainly consisting of excitatory glutamatergic projection neurons. Through widespread innervation on other basal ganglia components, STN provides a powerful driving force for the whole basal ganglia circuitry (Plenz and Kital, 1999). In addition, STN is not only a crucial node in the “indirect” fronto-striatal-pallidal-subthalamic pathway, but also forms the “hyperdirect” fronto-subthalamic pathway which directly connects the cortex (Nambu et al., 2002; Kravitz et al., 2010; Chu et al., 2015; Zhuang et al., 2018a). *Via* sending excitatory input to the internal globus pallidus, the STN balances the activity of the “direct” fronto-striatal-pallidal pathway and thus contributes to modulate an appropriate initiation and execution of voluntary movement. Lesion of the STNs leads to ballism (Barlas et al., 2001), a syndrome characterized by continuous, violent, involuntary, wild, and flinging movements of the proximal parts of the limbs. Moreover, a series of recent studies have documented that STN also holds a key position in action selection, response vigor, reinforcement learning, as well as cognitive, emotional, and motivational functions (Wagenbreth et al., 2015; Zavala et al., 2015; Dunovan and Verstynen, 2016; Zénon et al., 2016; Fischer et al., 2017).

Notably, orexinergic cell bodies are localized adjacent to the STN, which send a high-density of projections to the nucleus (Peyron et al., 1998; Sakurai et al., 1998), and even orexin mRNAs can be detected in the STN, indicating a modulatory role of orexin on STN neurons. Moreover, *in situ* hybridization and immunohistochemical studies have illustrated that the OX1/OX2 receptor mRNAs and proteins are expressed in the STN (Trivedi et al., 1998; Hervieu et al., 2001; Cluderay et al., 2002). By using *in vivo* electrophysiological recordings, the effect of orexin-A and orexin-B on STN neuronal firing rate has been reported recently (Sheng et al., 2018). However, the ionic mechanisms underlying the excitatory effect of orexin on STN neurons remain unknown. Therefore, in the present study, by using whole-cell patch clamp recording and immunostaining techniques, we showed that orexin directly excited STN neurons *via* postsynaptic OX1 and OX2 receptors, and the two orexin receptor subtypes co-expressed and co-localized on the same STN neurons. Also, a dual ionic mechanism involving both NCXs and inward rectifier K^+^ channels was found to mediate the orexin-induced excitation on STN neurons.

## Materials and Methods

### Animals

The experiments were conducted on Sprague-Dawley rats of either sex, housed under controlled conditions with a lighting schedule of 12 h light and 12 h darkness at 22 ± 2°C. Standard food and water were provided *ad libitum*. Rats aged about 14–21 days were used for whole-cell patch clamp recordings since the central orexinergic system seems to reach adult-like levels around P21 in rats and change only slightly in the post-weaning development in Eastern gray kangaroos (Steininger et al., 2004; Yamamoto et al., 2006). Rats aged about 8 weeks were used for immunohistochemical study. All animal experiments were approved by the Experimental Animal Care and Use Committee of Nanjing University and were conducted in accordance with the US National Institutes of Health Guide for the Care and Use of Laboratory Animals (NIH Publication 85-23, revised 2011). All efforts were made to minimize the number of animals used and their suffering.

### Whole-Cell Patch Clamp Recordings

**Figure 1:**
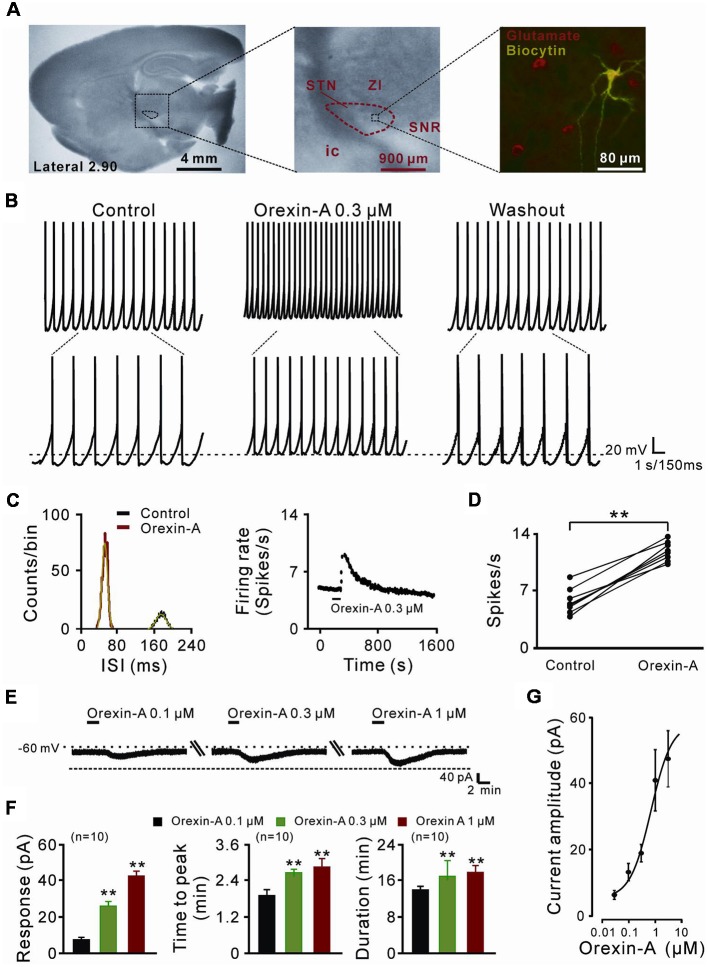
The direct excitatory effect of orexin on the subthalamic nucleus (STN) neurons. **(A)** Microscope image of a STN which centrally located in a 300 μm thick brain sagittal slice (observed with Olympus BX51WI, using a 40× water immersed objective) and a glutamatergic STN neuron labeled with biocytin after patch-clamp recording. **(B)** Orexin-A (300 nM) excited a STN spontaneous firing neuron in current clamp recording. **(C)** Orexin changed the distribution of inter-spike intervals (the red curve is Gaussian fit to the data) and increased firing rate of the STN neuron presented in **(B)**. **(D)** Group data of the effect of orexin-A on firing rate of STN neurons (*n* = 8). **(E)** Orexin-A concentration-dependently elicited the inward current and increased time to peak and duration of response of the recorded STN neuron. **(F)** A group of data recorded from 10 STN neurons. **(G)** Concentration-response curve for orexin-A on STN neurons show mean EC50 value of 29.0 ± 14.3 nM (*n* = 8). Data are presented as mean ± SEM; **P < 0.01. In this and the following figures, the short horizontal bars above the experimental records indicate the 1 min period of application of orexin-A, and the long horizontal bars indicate the exposure of the slice to tetrodotoxin (TTX), antagonists or blockers of receptors, ion exchangers or channels.

Whole-cell patch clamp recordings were performed as previously described (Zhang et al., 2011; Wang et al., 2017; Zhuang et al., 2018a,b; Ji et al., 2019) on STN neurons on brain slices to assess the effect of orexin and the underlying receptor and ionic mechanisms. According to the rat brain atlas of Paxinos and Watson (2014), sagittal slices (300 μm in thickness) containing the STN were cut (Figure 1A) with a vibroslicer (VT 1200S, Leica) at 4°C. The slices were incubated in artificial cerebrospinal fluid (ACSF, composition in mM: 124 NaCl, 2.5 KCl, 1.25 NaH_2_PO_4_, 1.3 MgSO_4_, 26 NaHCO_3_, 2 CaCl_2_ and 10 D-glucose) equilibrated with 95% O_2_ and 5% CO_2_ at 35°C ± 0.5°C for at least 1 h and then maintained at room temperature. During recording sessions, the slices were transferred to a submerged chamber and continuously superfused with 95% O_2_ and 5% CO_2_ oxygenated ACSF at a rate of 2 ml/min maintained at room temperature.

Whole-cell recordings were performed on STN neurons with borosilicate glass pipettes (3–5 MΩ) filled with an internal solution (composition in mM: 135 K-methylsulfate, 5 KCl, 2 MgCl_2_, 10 HEPES, 5 EGTA, 0.5 CaCl_2_, 4 Na_2_-ATP, 0.4 GTP-Tris, adjusted to pH 7.25 with 1 M KOH). Recordings of STN neurons were carried out in slices that were superfused with ACSF. STN neurons were visualized with an Olympus BX51WI microscope (Olympus, Tokyo, Japan) equipped with infrared differential interference contrast. Patch-clamp recordings were acquired with an Axopatch-700B amplifier (Axon Instruments, Sunnyvale, CA, USA) and the signals were fed into a computer through a Digidata-1440A interface (Axon Instruments) for data capture and analysis (pClamp 10.5, Axon Instruments). Neurons were held at a membrane potential of −60 mV and characterized by injection of rectangular voltage pulses (5 mV, 50 ms) to monitor the whole-cell membrane capacitance, series resistance, and membrane resistance. Neurons were excluded from the study if the series resistance was not stable or exceeded 20 MΩ.

We bathed the slices with orexin-A (0.03–3 μM, Tocris, Bristol, UK) to stimulate the recorded neurons. Tetrodotoxin (TTX, Alomone Labs, Israel), NBQX (AMPA/kainate receptor antagonist, 20 μM; Tocris), D-AP5 (NMDA receptor antagonist, 50 μM; Tocris) and gabazine (GABA_A_ receptor antagonist, 50 μM; Tocris) were used to examine the direct postsynaptic effect of orexin-A. SB334867 (10 μM, Tocris) and JNJ10397049 (10 μM, Tocris), high selective antagonists for OX1 and OX2 receptor respectively, were applied to assess the underlying receptor mechanism. Selective NCX blocker KB-R7943 (50 μM, Alomone Labs, Israel), broad spectrum K^+^ channel blocker BaCl_2_ (1 mM) and selective inward-rectifier K^+^ channel blocker tertiapin-Q (100 nM, Tocris) were used to explore the underlying ionic mechanism. Moreover, to determine the characteristic of whole cell current induced by orexin-A, in voltage-clamp recordings, current-voltage plots (*I*-*V curves)* were obtained before and during application of orexin-A using a slow ramp command (*dV*/*dt* = −10 mV/s, ranged from −60 to −120 mV) to allow for attainment of steady-state conditions.

### Immunohistochemistry and Imaging

The experimental procedures for immunostaining followed our previous work (Li et al., 2017; Wang et al., 2017; Zhuang et al., 2018a,b). Rats were deeply anesthetized with sodium pentobarbital (65 mg/kg) and perfused transcardially with 100 ml normal saline, followed by 250–300 ml 4% paraformaldehyde in 0.1 M phosphate buffer. Subsequently, the brain was removed, trimmed and postfixed in the same fixative for 12 h at 4°C, and then cryoprotected with 30% sucrose for 48 h. Frozen coronal sections (25 μm thick) containing the STN were obtained by using a freezing microtome (CM1860, Leica, Wetzlar, Germany) and mounted on gelatin-coated slides. The slices were rinsed with PBS containing 0.1% Triton X-100 and then incubated in 10% normal bovine serum in PBS containing 0.1% Triton X-100 for 30 min. Sections were incubated overnight at 4°C with primary antibody/antibodies, as follows: mouse anti-glutamate (1:1,000; Millipore Sigma, St. Louis, MO, USA; Cat# MAB5304, RRID:AB_94698), chicken anti-OX1 receptor (1:200; Acris Antibodies GmbH, Germany; Cat# BP4012, RRID:AB_1005881), rabbit anti-OX2 receptor (1:100; Millipore Sigma; Cat# AB3094, RRID:AB_91358). After a complete wash in PBS, the sections for single or double immunostaining were incubated in the related secondary antibodies (1:2,000; Life Technologies Carlsbad, CA, USA) conjugated to Alexa Fluor 488 and/or Alexa Fluor 594 (1:2,000, Life Technologies) for 2 h at room temperature in the dark. The slides were washed and mounted in Fluoromount-G mounting medium (Southern Biotech, Birmingham, AL, USA). Incubations replacing the primary antiserum with control immunoglobulins and/or omitting the primary antiserum were used as negative controls. All micrographs were taken with an inverted laser scanning confocal FluoView FV1000 microscope (Olympus, Tokyo, Japan), equipped with Plan-Apochromat ×60/1.42 NA oil, ×40/0.9 NA dry, ×20/0.75 NA dry, and ×10/0.4 NA dry objective lenses. Digital images from the microscope were recorded with FV10-ASW 3.1 Viewer Software (Olympus) and image processing was done with Photoshop (Adobe Systems Inc., San Jose, CA, USA).

### Statistics

All data were analyzed with SPSS 17.0 (SPSS, Chicago, IL, USA) and presented as mean ± SEM Shapiro-Wilk test was used for testing the normality of data. Two-tailed Student’s *t*-test was employed for statistical analysis. *P*-values of < 0.05 were considered to be significant.

## Results

### Orexin-A Excites STN Neurons *via* a Direct Postsynaptic Effect

In the present study, a total of 70 STN neurons with the input resistance higher than 150 MΩ were recorded. All the glutamatergic neurons we patched had a diameter >25 μm (Figure 1A) and showed spontaneous firing, which was in agreement with the previous reports (Baufreton et al., 2005; Atherton et al., 2010). In current clamp experiments, brief bath application of orexin-A (0.3 μM, 1 min) elicited a significant increase in the discharge rate of STN neurons (8/11, 72.72%) from 6.0 ± 0.5 spikes/s to 12.0 ± 0.4 spikes/s (*n* = 8, *P* < 0.01; Figures 1B,D). The inter-spike intervals and peri-stimulus time histograms showed that orexin-A largely changed the distribution of inter-spike intervals and increased the firing rate of the STN neurons (Figure 1C). On the other hand, in voltage-clamp experiments, 0.1, 0.3, and 1 μM orexin-A induced an inward current (from 7.2 ± 3.7 pA to 24.6 ± 5.2 pA and 43.5 ± 4.8 pA, respectively, *n* = 10, *P* < 0.001, respectively), increased the time to peak (from 1.8 ± 0.6 min to 2.5 ± 0.2 min and 2.7 ± 0.8 min, respectively, *n* = 10, *P* < 0.01, respectively), and prolonged the duration of response (from 14.8 ± 0.3 min to 17.3 ± 1.0 min and 17.9 ± 0.4 min, respectively, *n* = 10, *P* < 0.01, respectively) on STN neurons in a dose-dependent manner (Figures 1E,F). Fitting the concentration-response curve from eight STN neurons yielded that the concentration of orexin-A required for half-maximal activation (EC_50_) was 28.3 ± 11.6 nM (Figure 1G).

**Figure 2:**
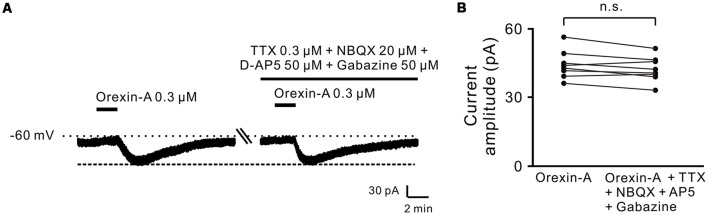
Orexin-A excited the recorded STN neurons with a postsynaptic manner. **(A)** TTX, NBQX, D-AP5 and gabazine did not block the inward currents induced by orexin-A on a recorded STN neuron. **(B)** Group data of the recorded STN neurons (*n* = 8). Data are presented as mean ± SEM; n.s., no statistical difference.

Moreover, combined application of TTX (0.3 μM), NBQX (20 μM, a potent AMPA receptor antagonist), D-AP5 (50 μM, a potent NMDA receptor antagonist) and SR95531 (50 μM, a GABA_A_ receptor antagonist) did not block the orexin-A-elicited inward current on the recorded neurons (44.3 ± 2.1 pA vs. 42.3 ± 1.9 pA, *n* = 8, *P* > 0.05; Figures 2A,B), strongly indicating that orexin excites STN neurons *via* a direct postsynaptic effect.

### Orexin-A Excites STN Neurons by Activation of Both OX1 and OX2 Receptors

**Figure 3:**
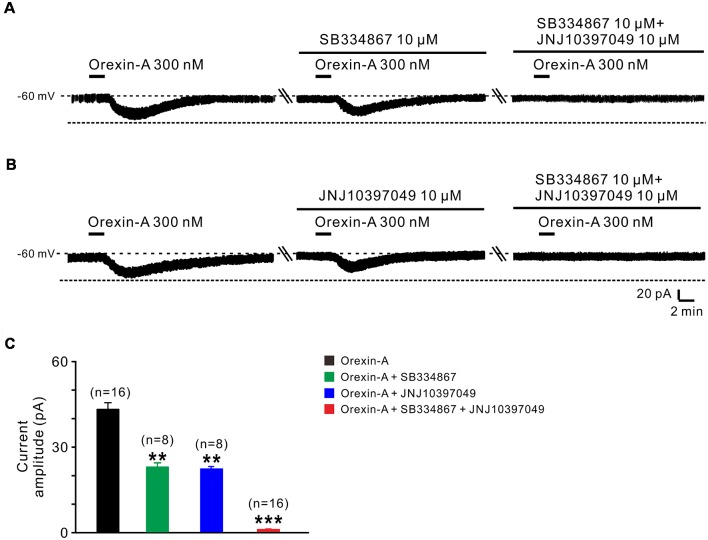
OX1 and OX2 receptors co-mediate the excitation of orexin on STN neurons. **(A)** Orexin-A (300 nM) elicited an inward current in a STN neuron, SB334867 (10 μM), a selective antagonist for OX1 receptor, partly blocked the current induced by orexin-A and SB334867 combined with JNJ10397049, a selective antagonist for OX2 totally abolished the orexin-A-induced inward current. **(B)** Orexin-A (300 nM) elicited an inward current in a STN neuron, JNJ10397049 (10 μM) partly blocked the current induced by orexin-A and JNJ10397049 combined with SB334867 totally abolished the orexin-A-induced inward current. **(C)** Group data of the tested STN neurons under orexin-A induced inward current as present in (**A**, *n* = 8) and (**B**, *n* = 8). Data are presented as mean ± SEM, **P < 0.01, ***P < 0.001.

Orexin-A exerts its physiological actions *via* two G protein-coupled orexin receptors, OX1 and OX2 receptor (Sakurai et al., 1998; Marcus et al., 2001). Therefore, in the present study, we used SB334867 (selective OX1 receptor antagonist) and JNJ10397049 (selective OX2 receptor antagonist) to examine which receptor(s) mediated the orexin-induced excitation on STN neurons (Figure 3). The orexin-A-elicited inward current was partly blocked by separate application of SB334867 (10 μM; from 44.5 ± 2.5 pA to 23.6 ± 1.4 pA, *n* = 8, *P* < 0.01; Figures 3A,C) or JNJ10397049 (10 μM; from 44.6 ± 2.5 pA to 22.6 ± 0.5 pA, *n* = 8, *P* < 0.01; Figures 3B,C). Moreover, combined application of SB334867 and JNJ10397049 nearly totally antagonized the orexin-A-induced excitation from 44.6 ± 2.5 pA to 1.2 ± 0.1 pA on STN neurons (*n* = 16, *P* < 0.001; Figures 3A–C). All these results suggest that OX1 and OX2 receptors co-mediate the excitatory effect induced by orexin-A on STN neurons.

**Figure 4:**
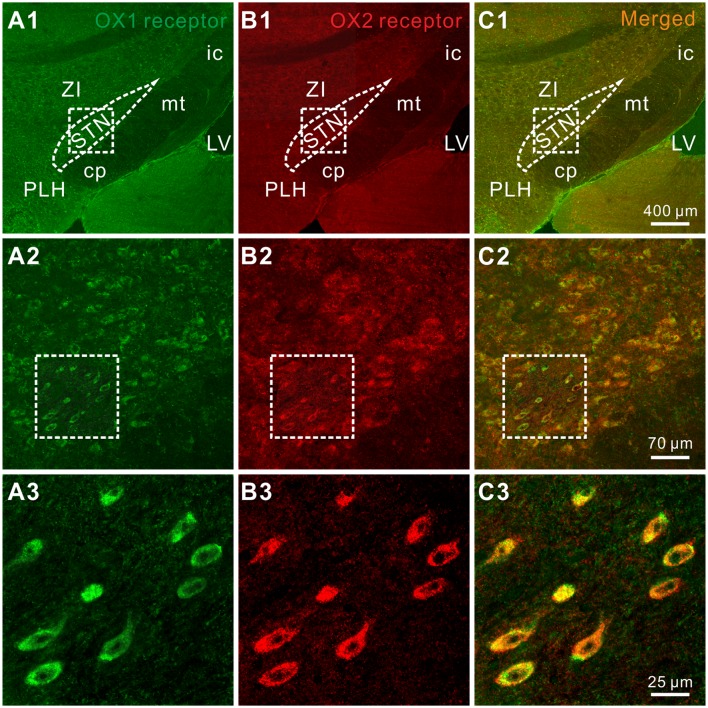
Double-labeled immunofluorescence staining for OX1 (green) and OX2 (red) receptors in rat STN. **(A1–A3)** OX1 receptor staining. **(B1–B3)** OX2 receptor staining. **(C1–C3)** Merged images showing colocalization of OX1 and OX2 receptors in the same STN neurons. STN, subthalamic nucleus; ZI, zona incerta; 3V, 3th ventricle; 4V, 4th ventricle; cp, cerebral peduncle; ic, internal capsule; mt, mammillothalamic tract; PLH, peduncular part of the lateral hypothalamus.

Furthermore, the distribution of OX1 and OX2 receptors was mapped in the STN by double immunofluorescence staining. We found that for all the stained sections (five rats and 10 sections for each) both of the orexin receptor subtypes were not only co-expressed in the STN (Figures 4A1–A3,B1–B3) but also co-localized in the same neurons (Figures 4C1–C3), which was consistent with the electrophysiological results mentioned above.

### Orexin-A Excites the STN Neurons *via* Activation of NCXs and Closure of Inward Rectifier K^+^ Channels

**Figure 5:**
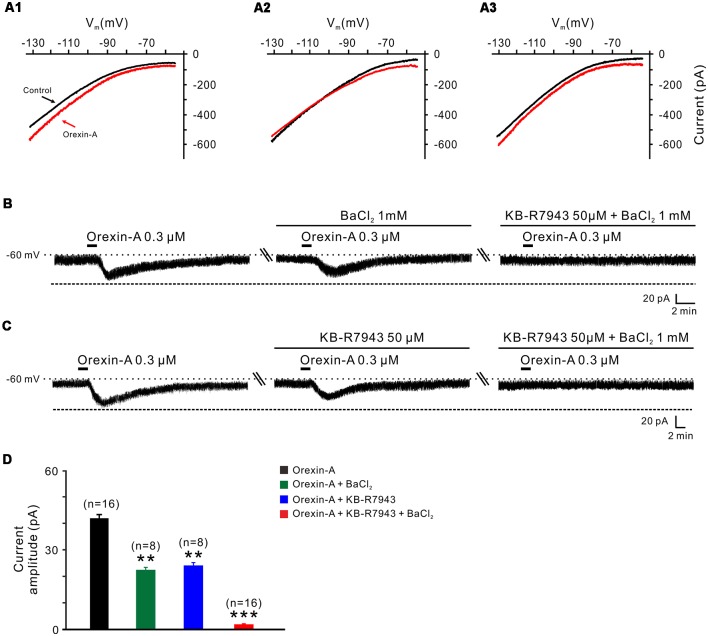
Na^+^-Ca^2+^ exchangers (NCXs) and K^+^ channels co-mediate the excitation of orexin on STN neurons. **(A1–A3)** *I*-*V* relationships of STN neurons in the absence and presence of orexin. In 63.8% of the neurons tested, the orexin A-induced inward current was larger at the more hyperpolarized potential of −130 mV than at −55 mV **(A1)**; in 22.4% of these neurons tested, the orexin A-induced inward current reversed near the calculated Ek of −105 mV **(A2)**; in 13.8% neurons, the orexin A-induced inward current first decreased then increase amplitude along with the holding potential hyperpolarization, and was similar in magnitude at −55 and −130 mV **(A3)**. **(B)** Orexin-A (300 nM) elicited an inward current in a STN neuron. BaCl_2_, a broad spectrum blocker of K^+^ channels, partly blocked the effect of orexin-A on STN neurons and combined application of the NCX blocker KB-R7943 totally abolished the orexin-A-induced inward current (*n* = 8). **(C)** Orexin-A (300 nM) elicited an inward current in a STN neuron. KB-R7943 partly blocked the effect of orexin-A on STN neurons and combined application of the BaCl_2_ totally abolished the orexin-A-induced inward current (*n* = 8). **(D)** Group data of the 16 tested STN neurons under orexin-A induced inward current as present in **(B,C)**. Data are presented as mean ± SEM, **P < 0.01, ***P < 0.001.

Next, we applied slow-ramp command tests and determined the *I*-*V* curves in response to orexin-A to investigate the underlying ionic mechanisms of orexin on STN neurons. We observed three types of the orexin-A-induced changes on the *I*-*V* curves from STN neurons (*n* = 15; Figures 5A1–A3). The diversity of the orexin-A-induced changes in *I*-*V* relationships implies that more than one ionic mechanism is involved in the orexin-A-induced excitation on STN neurons. In 8 of 15 neurons, the *I-V* curves in the absence and presence of orexin-A were apart more at −130 mV as compared with −55 mV, indicating that ion channels/exchangers with the reversal potential depolarized than −60 mV may be involved in the orexin-A-induced net current (Figure 5A1). Considering NCXs were reported to be coupled to orexin receptors in many different brain regions and have a more positive reversal potential (Wu et al., 2004; Zhang et al., 2011), we thus speculated that the activation of NCXs may mediate the orexin-induced change in the *I*-*V* relationships. Furthermore, in 5 of 15 recorded STN neurons, the *I*-*V* curves in the absence and presence of orexin-A intersected at the −105 mV (Figure 5A2), which means that the orexin-A-induced inward current reverses near the calculated E_k_ of −105 mV, thus indicating that K^+^ channels may be involved in the effect of orexin-A on STN neurons. In the remaining two neurons, the orexin-A-elicited change in the *I*-*V* curves was similar in amplitudes at −55 and −130 mV (Figure 5A3), although the amplitude first decreased then increased along with the hyperpolarization.

To further confirm the results of slow-ramp command tests, we applied Ba^2+^ (a broad spectrum blocker for K^+^ channels) and KB-R7943 (a potent and selective inhibitor for NCXs) to determine whether K^+^ channels and NCXs are involved in the effect of orexin-A on STN neurons. We found a partial inhibition of the orexin-A-induced inward current either by Ba^2+^ (1 mM; from 41.0 ± 1.3 pA to 22.2 ± 0.5 pA, *n* = 8, *P* < 0.01; Figures 5B,D) or by KB-R7943 application (50 μM; from 42.5 ± 1.7 pA to 24.5 ± 0.7 pA, *n* = 8, *P* < 0.01; Figures 5C,D). Moreover, the orexin-A-induced inward current was totally blocked from 41.8 ± 1.5 pA to 1.6 ± 0.2 pA by combined application of Ba^2+^ and KB-R7943 (*n* = 16, *P* < 0.001; Figures 5B–D), suggesting that the closure of K^+^ channels as well as activation of NCXs co-mediated the excitation of orexin-A on STN neurons.

**Figure 6:**
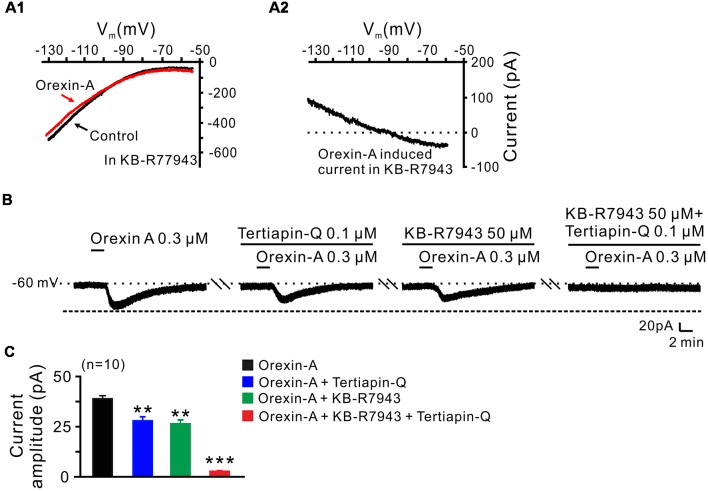
Inward rectifier K^+^ channels and NCXs contribute to the excitatory effect of orexin on STN neurons. **(A1,A2)** *I*-*V* relationship shows an outward rectifier K^+^ current was exposed after KB-R7943 inhibited the activation of the NCX. **(B)** Orexin-A (300 nM) elicited an inward current in a STN neuron. KB-R7943 partly blocked the effect of orexin-A on STN neurons and combined application of the inward rectifier K^+^ channel antagonist tertiapin-Q totally abolished the orexin-A-induced inward current. **(C)** Group data of the 10 tested STN neurons under orexin-A induced inward current as present in **(B)**. Data are presented as mean ± SEM, **P < 0.01, ***P < 0.001.

In order to clarify which kind of K^+^ channels contributes to the excitatory effect of orexin on STN neurons, we further analyzed the characteristics of the orexin-A-induced K^+^ current component. Under a condition of blockage of NCXs by continuously perfusing the slice with KB-R7943, we used slow ramp command tests to obtain the *I*-*V* curves in the absence and presence of orexin-A (Figures 6A1,A2). The results showed that the difference current had a reversal potential of −100 mV that was near the calculated E_k_ and exhibited a characterization of strongly outwardly rectifying (Figure 6A2). Since, the closure of K^+^ channels is responsible for depolarization, the result indicates that the K^+^ channels blocked by orexin-A are the inward rectifier K^+^ channels. As shown in Figures 6B,C, the orexin-A induced inward current on STN neurons was partly blocked by separate application of specific inward rectifier K^+^ channels antagonist tertiapin-Q (100 nM; from 49.3 ± 6.8 pA to 27.9 ± 3.8 pA, *n* = 10, *P* < 0.01; Figures 6B,C) or KB-R7943 (50 μM; from 49.3 ± 6.8 to 26.5 ± 4.6 pA, *n* = 10, *P* < 0.01; Figures 6B,C), and totally blocked by combined application of KB-R7943 and tertiapin-Q (from 49.3 ± 6.8 to 2.5 ± 0.6 pA, *n* = 10, *P* < 0.001; Figures 6B,C). All these results strongly indicate that the excitatory effect of orexin-A on STN neurons is mediated by a dual ionic mechanism including both activation of the NCXs and blockage of the inward rectifier K^+^ channels.

## Discussion

As a driving force for the integrated function of basal ganglia circuitry, the STN plays a key role in the motor initiation and execution. However, little is known about the endogenous factors modulating STN neuronal activity. In the present study, we report that orexin, a hypothalamic neuropeptide, directly excites STN neurons *via* postsynaptic OX1 and OX2 receptors. And a dual ionic mechanism including activation of the NCXs and closure of the inward rectifier K^+^ channels mediates the excitatory effect of orexin-A on STN neurons.

Previous studies from our laboratory and others have revealed an extensive regulation of orexin on the neuronal activity within the basal ganglia nuclei. It has been documented that there is a selective excitation of orexin-A on the GABAergic neurons in the substantia nigra pars reticulata instead of the dopaminergic neurons in the substantia nigra pars compacta (Korotkova et al., 2002). Moreover, orexin-A directly enhances the excitability of globus pallidus internus neurons and ventral pallidal GABAergic neurons by direct activation of OX1 and OX2 receptors (Gao et al., 2016; Ji et al., 2019). However, in the striatum, instead of a direct postsynaptic effect, orexin-A potentiates the AMPA-mediated synaptic transmission on the corticostriatal synapses (Shin et al., 2009). In this study, we demonstrate an excitatory action of orexin on neurons in the STN *via* postsynaptic OX1 and OX2 receptors, which is in accordance with the previous neuropharmacological studies *in vivo*, previous and present immunohistochemical studies as well as the *in situ* hybridization on the distribution of orexinergic fibers and receptors (Peyron et al., 1998; Trivedi et al., 1998; Hervieu et al., 2001; Cluderay et al., 2002; Sheng et al., 2018). These results suggest that the central orexinergic system may modulate the major components in the basal ganglia circuitry in parallel and subsequently participate in regulation of motor behaviors, such as biased swing behavior (Sheng et al., 2018).

Several types of ionic channels/exchangers including K^+^ channels, nonselective cation channels and/or electrogenic NCXs have been reported to be linked to orexin receptors (Lytton, 2007; Kukkonen, 2011; Kukkonen and Leonard, 2014; Ji et al., 2019). *In situ* hybridization and immunocytochemical studies have revealed the distribution of NCX and inward rectifier K^+^ channel mRNAs in the basal ganglia (Karschin et al., 1994; Murer et al., 1997; Canitano et al., 2002; Jeon et al., 2008). Here, we find that both the NCXs and inward rectifier K^+^ channels are involved in the excitation of STN neurons induced by the activation of orexin receptors. Because of the highly positive reversal potential (Wu et al., 2004), NCXs activation can provide a powerful force for neuronal depolarization. On the other hand, by extruding Ca^2+^ from the cytoplasm, NCXs prevent Ca^2+^ overload in the highly excited neurons. Nevertheless, distinct from the NCXs, the activation of inward rectifier K^+^ channels are responsible for the repolarization of membrane action potentials, and their shutoff help to generate a spike (Hille, 2001; Nishida and MacKinnon, 2002). Thus, through activation of NCXs and closure of inward rectifier K^+^ channels, orexin strongly depolarizes and increases the discharge of spontaneous firing STN neurons. We speculate that *via* the dual ionic mechanism, orexin/central orexinergic system may subsequently bias the excitability of the STN neurons and actively participate in the regulation of STN mediated motor control, action selection, response vigor, reinforcement learning, as well as other cognitive, emotional, and motivational functions (Wagenbreth et al., 2015; Zavala et al., 2015; Dunovan and Verstynen, 2016; Zénon et al., 2016; Fischer et al., 2017).

The STN holds a key position not only in normal function of the basal ganglia but also in the pathological processes of basal ganglia disorders (Wang et al., 2018). A change in firing rate and/or firing patterns as well as an excess of neuronal synchronization of STN is a well-recognized hallmark in the parkinsonian state, and lesions of the STN have been reported to alleviate the parkinsonian motor symptoms (Bergman et al., 1990; Limousin et al., 1998; Rodriguez et al., 1998). Here, we report a direct excitation of orexin on STN neurons *via* both OX1 and OX2 receptors and the coupled NCXs and inward rectifier K^+^ channels. Intriguingly, parkinsonian patients show an increasing loss of orexin cells with disease progression and a decrease in orexin level in the cerebrospinal fluid (Fronczek et al., 2007; Thannickal et al., 2007). Therefore, the role of the central orexinergic system in the basal ganglia circuitry including the STN, especially the contribution of endogenous orexin to the basal ganglia motor functions and dysfunctions, needs to be further assessed. The ion channels and exchangers coupled to orexin receptors in the STN might be potential effective targets for the treatment of basal ganglia motor diseases such as Parkinson disease.

## Ethics Statement

All animal experiments were approved by the Experimental Animal Care and Use Committee of Nanjing University and were conducted in accordance with the US National Institutes of Health Guide for the Care and Use of Laboratory Animals (NIH Publication 85-23, revised 2011). All efforts were made to minimize the number of animals used and their suffering.

## Author Contributions

G-YL, Q-XZ and X-YZ performed experiments, analyzed data, and prepared figures and the draft manuscript. J-NZ and J-JW designed research and wrote the article.

## Conflict of Interest Statement

The authors declare that the research was conducted in the absence of any commercial or financial relationships that could be construed as a potential conflict of interest.
